# Insulin-Like Growth Factor I (IGF-1) Ec/Mechano Growth Factor – A Splice Variant of IGF-1 within the Growth Plate

**DOI:** 10.1371/journal.pone.0076133

**Published:** 2013-10-11

**Authors:** Werner Schlegel, Adalbert Raimann, Daniel Halbauer, Daniela Scharmer, Susanne Sagmeister, Barbara Wessner, Magdalena Helmreich, Gabriele Haeusler, Monika Egerbacher

**Affiliations:** 1 Medical University of Vienna, Department of Paediatrics and Adolescent Medicine, Vienna, Austria; 2 Veterinary University Vienna, Institute of Anatomy, Histology and Embryology, Vienna, Austria; 3 University of Vienna, Centre for Sports Sciences and University Sports, Department of Sports and Exercise Physiology, Vienna, Austria; University of Jaén, Spain

## Abstract

Human insulin-like growth factor 1 Ec (IGF-1Ec), also called mechano growth factor (MGF), is a splice variant of insulin-like growth factor 1 (IGF-1), which has been shown *in vitro* as well as *in vivo* to induce growth and hypertrophy in mechanically stimulated or damaged muscle. Growth, hypertrophy and responses to mechanical stimulation are important reactions of cartilaginous tissues, especially those in growth plates. Therefore, we wanted to ascertain if MGF is expressed in growth plate cartilage and if it influences proliferation of chondrocytes, as it does in musculoskeletal tissues. MGF expression was analyzed in growth plate and control tissue samples from piglets aged 3 to 6 weeks. Furthermore, growth plate chondrocyte cell culture was used to evaluate the effects of the MGF peptide on proliferation. We showed that MGF is expressed in considerable amounts in the tissues evaluated. We found the MGF peptide to be primarily located in the cytoplasm, and in some instances, it was also found in the nucleus of the cells. Addition of MGF peptides was not associated with growth plate chondrocyte proliferation.

## Introduction

Linear growth is a tightly regulated process achieved by enchondral ossification at the growth plates of long bones. The coordination of recruitment, proliferation, hypertrophy and apoptosis of growth plate chondrocytes plays a crucial role to ensure physiological growth. Therefore, the architecture of this mechanically strained and lowly oxygenated tissue is regulated by complex signal loops, mechanical factors, the metabolic situation of the individual and probably other so far unknown mechanisms.

The central role of the insulin-like growth factor 1 (IGF-1) protein family in the control of linear growth has been shown in numerous *in vivo* and *in vitro* studies [1], [2], [3], [4]. The local expression of the *Igf-1* gene was shown to be crucial for the maintenance of normal growth rates and chondrocyte differentiation [5].

**Figure 1:**
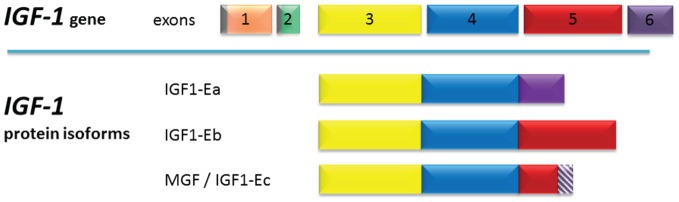
Genomic organization and protein structure of the porcine *Igf1*. The core transcript, consisting of exons 3 and 4 (yellow and blue), is either spliced to exon 1 (orange) or exon 2 (green). IGF1-Ea is joined to exon 6 (violet). IGF1-Eb is joined to exon 5 (red). *Mgf* consists of the exons 3, 4, the N-terminal part of exon 5, consisting of 49 or 52 nucleotides (depending on the species) and exon 6 (striated violet). Exon 5 evokes a frame shift in exon 6 resulting in an altered amino acid sequence at the C-terminal end. The two dark grey regions symbolize the promoter regions of the corresponding transcript. The colours of the exons were chosen to match a corresponding figure of the human IGF gene in Goldspink G Physiology 2005;20∶232–238.

The *Igf1* gene locus consists of 6 exons (Fig. 1). Exons 1 and 2 are individual leader exons with distinct promoter sequences. Either of the two initiation sites gives rise to insulin-like growth factor 1 (IGF-1) transcripts with specific signaling sequences [6], [7], [8]. The core IGF-1 protein is encoded by the exons 3 and 4 and is the mature form of the protein found in peripheral blood. The alternatively spliced exons 5 and 6 encode for the peptide domain E, which is present in IGF-1 precursor proteins [9]. Both the E-peptide and the signaling peptide are removed by protease cleavage, resulting in the mature IGF-1 protein [10], [11]. But not all of the IGF-1 peptide is secreted in its mature form; IGF-1 still connected to the E-peptide can also be detected outside of cells [12], [13], [14], [15].

Insulin-like growth factor genes appear to have evolved from a single insulin-like gene. This gene can be detected in invertebrates and seems to cause anti-apoptotic effects by maintaining terminally differentiated cells [16], [17], [18]. This progenitor for IGFs has diversified in vertebrates, generating a gene family with a considerable number of splice variants with different functions. Thus, the ancestral insulin-like gene has throughout its phylogeny given rise to a delicately regulated redundant, yet versatile, system, governing cell proliferation as well as differentiation.

Insulin-like growth factor 1 Ec (IGF-1Ec), also known as mechano growth factor (MGF), is a splicing variant of IGF-1. IGF-1Ec contains the core protein encoded by the exons 3 and 4, as well as a specific insert encoded by a region of exon 5. This 49 bp insert in humans introduces a reading frame shift, resulting in a different carboxy-terminal peptide sequence to that of IGF-1Ea (Fig. 1) [19], [20]. Several studies have suggested that this C-terminal peptide (corresponding to the Ec fragment) has a physiological function which is distinct from that of IGF-1 [19], [21], [22], [23]. IGF nomenclature varies and is species-specific. For clarity, we refer to this C-terminal peptide, which is derived from Ec, as MGF throughout the following text.

MGF has been found in many tissues. It has been reported to display a neuroprotective effect in cerebral regions which have been exposed to ischemia and to be expressed in stromal cells of the eutopic endometrium and in glandular cells of the ectopic endometrium [20], [24], [25].

IGF-1 and MGF are up-regulated in exercised and damaged skeletal muscle, probably inducing muscle growth and hypertrophy. MGF has been shown to stimulate proliferation and suppress differentiation, while IGF-1 also supported differentiation [19], [22], [23], [26], [27]. Similar observations concerning MGF have been made when cultured osteoblasts from newborn rat calvaria were exposed to mechanical stretch stimulation. Cyclic stretching of osteoblasts enhanced cell proliferation and induced expression of *Mgf* on the mRNA level [28]. MGF has been observed to inhibit osteoblast differentiation and mineralization in osteoblast cell culture medium [29]. In myoblasts, as well as in osteoblasts, the expression maximum of MGF is achieved before the systemic isoform reaches its expression maximum [18], [23]. MGF has been demonstrated to have a positive role on bone injury healing in an animal model of New Zealand white rabbits with 5-mm segmental bone defects inflicted by removal of the periosteum and endosteum in the middle of the radius. Injecting MGF into the bone defect gap for 5 consecutive days resulted in accelerated bone healing [30]. Noteable up-regulation of MGF expression could be observed in the Achilles tendons of Sprague Dawley rats exposed to hindlimb suspension for 7 days [31], [32]. Animal models have, therefore, confirmed cell culture experiments and identified MGF as a local tissue repair factor and also as a responder to mechanical demands.

Cartilage, although among the most prominent tissues exposed to recurrent mechanical demands, has not been investigated for expression of MGF. While articular cartilage is seen as terminally differentiated tissue and has a very limited capacity for regeneration, epiphyseal growth plate cartilage is highly dynamic and crucial for longitudinal growth as it orchestrates the complex processes of proliferation and differentiation. Adjacent tissues such as the groove of Ranvier are also involved in these processes [33]. The main isoforms of IGF-1 are known to play a key role in this regulatory system, but the effects of MGF on growth plate chondrocytes are yet to be explained [1], [2], [3], [4], [34], [35].

The aim of our study was to investigate whether MGF is present in the growth plate – a central *structure* for linear growth – and the extent to which Mgf mRNA contributes to the total amount of Igf1 mRNA – a central *hormone* for linear growth. Based on previous studies in other tissues showing the upregulation of MGF to initiate tissue regeneration and growth, we expected a substantial portion of total expression of IGF-1 isoforms in growth plate chondrocytes to be MGF.

Furthermore we wanted to explore if there is a proliferation effect on the growth plate when the C-terminal MGF peptide is exogenously added to growth plate chondrocytes in cell culture, as has been described for some other cell types. Revealing the role of MGF in chondrocyte differentiation and proliferation provides further insight into the complex local mechanisms of IGF-1 proteins to regulate linear growth.

## Materials and Methods

### Tissue sampling

We used piglets as our model system in our studies because pigs have a similar number of chondrocytes in the growth plate zones and similar cell kinetics to humans [36], [37], [38], [39], [40].

The methods of obtaining animals and tissues were evaluated and approved by the ethics committee of the Veterinary University of Vienna. All animal work was performed in dead animals and conducted according to the applicable Austrian guidelines. The piglets were a cross between Large White and Landrace breeds. They were killed at the Institute of Parasitology at the Veterinary University Vienna. Growth plates were collected from the distal femur and the proximal tibia of ten piglets killed at 3–6 weeks of age corresponding to the developmental stage of prepubertal children. The limbs were resected and the entire epiphysis was broken off from the femur and tibia at the level of the ossification front of the growth plate. Pieces of the growth plates were loosened from the bone by undermining with a scalpel. The growth plate pieces were transferred into formalin and fixed for at least 24 hours at room temperature for histology and immunohistochemistry, transferred into liquid nitrogen and then stored at −80°C (cryo-conservation) for use in laser microdissection (LMD), or transferred into chondrocyte culture medium (see below). In addition, samples of articular cartilage, groove of Ranvier, secondary center of ossification, skeletal muscle and tendons were collected from the respective piglets. Liver and brain tissues were used as control tissues.

### Histology and immunohistochemistry

Proximal tibia and distal femur growth plates from the piglets were dissected and fixed in 4% buffered formalin and embedded in paraffin. 4-µm sections were mounted on APES-glutaraldehyde-coated slides and dried at 37°C overnight. Immunohistochemical staining was carried out according to published protocols [41]. Briefly, after deparaffination, slides were blocked with H_2_O_2_ in methanol (0.6%) and incubated with goat serum. The primary antibodies rabbit anti-MGF serum (Phoenix Pharmaceuticals, Inc., Belmont, CA, USA, dilution 1∶500) and rabbit anti-IGF-1 (IBT Immunological& Biochemical Testsystems GmbH, Reutlingen, Germany, dilution 1∶50) were applied overnight at 4°C. We used anti-rabbit PowerVision HRP (ImmunoVision Technologies, Brisbane, CA, USA) with a substrate of DAB as secondary systems.

Immunofluorescence for MGF and IGF1 was performed on growth plate sections in order to differentiate nuclear versus cytoplasmic staining using anti-rabbit Alexa fluor 488 (green, Molecular Probes) as secondary Ab and DAPI (blue) for nuclear staining.

Negative controls were prepared by i) omission of the primary antibody but applying the secondary systems and ii) specific peptide blocking by pre-incubation of the respective antibody with the MGF C-terminal peptide (Phoenix Pharmaceuticals, Inc., Belmont, CA, USA) and IGF-1 peptide (ProSpec-Tany TechnoGene, Rehovot, Israel) using an antibody to peptide ratio of 1∶10 (Fig. S1).

### Laser microdissection

Cryo samples were cut into 6-µm thick sections at a temperature of −15°C using the Leica 1800 CM cryostat. All sections to be used for laser microdissection were mounted on special metal frame slides covered with a polyethylene naphtalate membrane (MMI, Glattbrugg, Switzerland). The LMD slides with the tissue sections were stained with the HistoGene Frozen Section Staining Kit from Arcturus (Molecular Devices, MDS Analytical Technologies GmbH, Ismaning, Germany). The staining process was carried out according to the manufacturer's instructions.

The sections were dried immediately for approximately 10 minutes (for reserve zone) or the chondrocyte matrix was removed from all but one section with a needle before the drying process (for proliferative and hypertrophic zones). The Veritas Microdissection System by Arcturus Engineering was used in this study. The energy of the cutting laser was set at to 11 (arbitrary unit on scale 0–32, maximum energy 4 mW), the power of the capture laser was 70 mW and the pulse of the capture laser was set at 2500 µs. The chondrocytes of each zone were collected on CapSure Macro caps (MDS) and the cap placed onto a tube containing the extraction buffer from the Qiagen RNeasy Micro Kit. The cells were lysed into the buffer at room temperature for 30 minutes and were centrifuged for 2 minutes at maximum speed after the incubation period. Chondrocytes from 18 sections were pooled for each zone. The cell extracts were frozen at −80°C.

### Extraction and purification of total RNA from chondrocytes and control tissues

Porcine chondrocytes were homogenized by mechanical disruption of the frozen tissue (liquid nitrogen) using a mortar and pestle. RNA was isolated as described in the RNeasy Mini Handbook (Qiagen, Hilden, Germany, 06/2001). Cells from monolayer culture were harvested by adding 1 ml of the TRI reagent kit (Sigma-Aldrich, St. Louis, MO, USA). RNA was isolated according to the manufacturer's instructions. The purity and amount of RNA were determined by measuring the OD260∶280 ratio.

### cDNA synthesis

1 μg of the total RNA was diluted with nuclease-free water to a volume of 15 μl. 4 μl of iScript™ reaction mix and 1 μl of iScript reverse transcriptase were added (Bio-Rad Laboratories, Hercules, CA, USA) to the RNA solution. The mix was incubated for 5 minutes at 25°C and for 30 minutes at 42°C. The cDNA synthesis was stopped by heating the reaction at 85°C for 5 minutes. The reaction was diluted by adding 20 μl (LMD) or 80 μl of nuclease-free water.

### Primers and probes for quantitative analyses

Primers and probes were designed using the Primer3 program (http://frodo.wi.mit.edu/primer3) to create oligo nucleotides with similar melting temperatures and minimal self-complementarity. The probes for the detection of total *Igf1* were placed at the junction of exons 3 and 4 to avoid amplification of genomic DNA. The primers for the amplification of the porcine *Mgf* transcript were placed at the junction of exons 4 and 5 (NCBI Nucleotide, Accession number (CN157588), nucleotide 501–520, forward primer) and the junction of the truncated exons 5 and 6 (NCBI Nucleotide, Accession number (CN157588), nucleotide 549–571, reverse primer), generating a product size of 71 bp. The gene specificity of the primers and probes and absence of DNA polymorphism were confirmed by BLASTN searches. Primers and probes were synthesized from GenXpress (Wiener Neudorf, Austria). Primer concentrations were tested for each primer at concentrations of 50 nM, 300 nM, and 900 nM, choosing the combination that displayed the lowest Ct value. The similar PCR reaction efficiencies allowed comparison of the expression levels of the different genes evaluated. Primer sequences are shown in Table 1.

**Table 1:** Primer sequences for real-time PCR.

Primers (Accession number)		Sequence	Primer Efficiency
Pig *Mgf*	FWD	CAGAAGTATCAGCCCCCATC	0.86
(CN157588)	REV	TCAAATGTACTTCCTTTCCTTCG	
	HYB	CCAACAAGAAAACGAAGTCTCAGAGG	
Pig *Igf1*	FWD	GTTCGTGTGCGGAGACAGG	0.83
(DQ784687)	REV	GCCCTCCGACTGCTGGA	
	HYB	CTTTTATTTCAACAAGCCCACAGGGTACGG	

### Real-time PCR amplification and analysis

The mRNA was quantified using real-time PCR. PCR amplification was performed and monitored with a 7500 fast real-time PCR system (Applied Biosystems, Foster City, CA, USA). The master mix was based on the 2× SensiMix dU DNA Kit (Quantance, London, UK) using a final Mg^2+^ concentration of 5.5 mM. Thermal cycling conditions comprised the initial steps at 50°C for 2 minutes followed by 95°C for 10 minutes. The cDNA products were amplified with 40 PCR cycles, consisting of a denaturation step at 95°C for 15 seconds, and annealing and extension steps at 60°C, each for 1 minute. All probes were normalized to 18S rRNA using the pre-developed Taqman assay (Applied Biosystems, California, USA). All cDNA samples (2.4 µl in 20 µl) were analyzed in triplicate. The final numeric value was calculated by the ΔΔCT method using a calibrator based on previous data on total IGF-1 expression in laser capture microdissected resting zone chondrocyte samples (12.86) [42]. The resulting values were expressed in arbitrary units.

### Primary cell culture

Growth plate cartilage was collected in a medium containing DMEM and 10% FCS. The samples were incubated for 30 minutes in an antibiotic solution consisting of PBS, 5 µg/ml amphotericin B and 200 µg gentamycin. Afterwards, they were cut into small pieces and incubated for at least 1 day in DMEM containing 236U/ml collagenase II (Gibco, Carlsbad, CA, USA), 2 µg/ml amphotericin B and 100 µg/ml gentamycin. The separated cells were filtered through a 40-µm filter and collected by centrifugation. Isolated chondrocytes were propagated in monolayer cell culture as described [43]. The chondrocyte culture medium consisted of DMEM containing 4 mM l-glutamine, 2 mg/l amphotericin B, 5 mg/l insulin, 50 mg/l ascorbic acid 2-phosphate Mg salt hydrate, 100 mg/l gentamycin and 10% FCS. Cells were cultivated at 37°C under 5% CO_2_.

### Effect of MGF on growth plate chondrocyte proliferation

Monolayer cells were cultivated for 1 week in chondrocyte culture medium (see 2.8) at 37°C under 5% CO_2_. 20,000 cells per cm^2^ growth area were transferred into 96-well plates containing serum-free stimulation medium for 48h. The stimulation medium consisted of a modified medium used by Benya and Loeser for chondrocyte growth factor studies [44], [45]: Phenol red-free DMEM containing 1 mM sodium pyruvate, 4 mM L-glutamine, 2 mg/l transferrin, 2 µg/l selenious acid, 420 mg/l BSA, 2.1 mg/l linoleic acid, 50 mg/l ascorbic acid, 2 µg/ml amphotericin B, and 100 mg/l gentamycin.

We treated chondrocytes with different variants of MGF C-terminal peptide. The effects of a consensus sequence unmodified C-terminal peptide MGF (Phoenix Pharmaceuticals, Karlsruhe, Germany) and of a synthetic, modified MGF peptide (referred as “modified MGF, Novabiochem, Nottingham, UK) were investigated. In contrast to the consensus sequence MGF, which has been reported to be degraded rapidly in body fluids, the modified MGF exhibited increased stability due to pegylatation and replacement of a L-arginine with a D-arginine (sequences shown in Table 2) [20].

**Table 2:** MGF Peptide sequences.

Unmodified peptide (consensus sequence)	NH2-YQPPSTNKNTKSQRKGSTFEEHK-COOH
Modified peptide [20]	NH2-YQPPSTNKNTKSQ (d) R (d) RKGSTFEEHK-COOH

Unmodified human MGF and a modified MGF peptide were used in concentrations of 0.1 to 500 ng/ml (0.1 ng/ml, 1 ng/ml, 3 ng/ml, 10 ng/ml, 50 ng/ml, 500 ng/ml) medium alone or combined with the same concentration of human IGF-1 (ProSpec-Tany TechnoGene, Rehovot, Israel). Cells treated with scrambled peptide (Phoenix Pharmaceuticals, Karlsruhe, Germany) were used as negative controls. Cells were harvested after incubation in the stimulation medium for 2 days and 4 days.

Cell proliferation was determined by the incorporation of 5-bromo-2′deoxyuridine (BrdU) using a BrdU labeling and detection kit (Roche Diagnostics, Mannheim, Germany). Procedures were as prescribed by the manufacturer.

### Statistical analysis

Data or log-transformed data were checked for normal distribution using the Shapiro-Wilk test. When data were normally distributed, a one-way ANOVA was used to determine differences between groups. Homogeneity of variances was proven by Levene's test, and post-hoc analyses were performed either by the Bonferroni or Games-Howell test. When data were not normally distributed, a Kruskal-Wallis test with manual Bonferroni correction (p<0.05/number of comparisons) was applied. In general the level of statistical significance was set at p<0.05. Data are shown as mean ± SEM. Statistical analyses were performed using the software package PASW Statistics (Version 18.0.0, Chicago, IL).

## Results

### Identification of the porcine *Mgf* sequence

**Figure 2:**
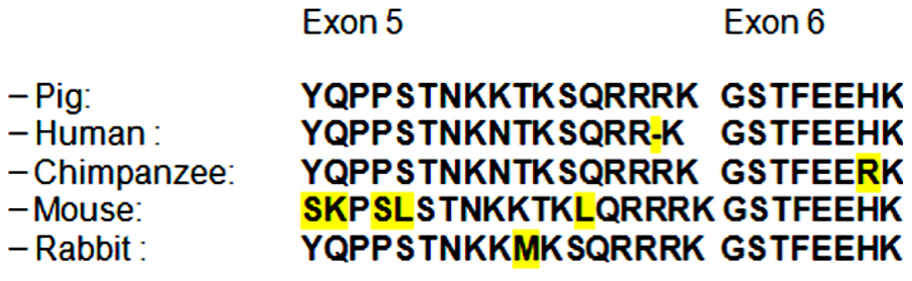
Peptide sequences of MGF in different species. MGF sequences are highly conserved and always consist of 25 amino acids with the exception of the human MGF peptide, which consists of only 24 amino acids.

Several groups have detected splicing variants of *Igf1* in different species, but not all mRNAs are fully covered. We performed comparative sequence analyses and found that one single complete clone of the porcine *Mgf* had already been sequenced but up until now had not been identified as this expression product (946304 MARC 4PIG Sus scrofa cDNA 5-, mRNA sequence). This sequence is available under the accession number CN157588. Comparison of *Mgf* sequences of different species showed high homology of the peptide sequence (Fig. 2).

### Expression of *Mgf* in selected porcine tissues

**Figure 3:**
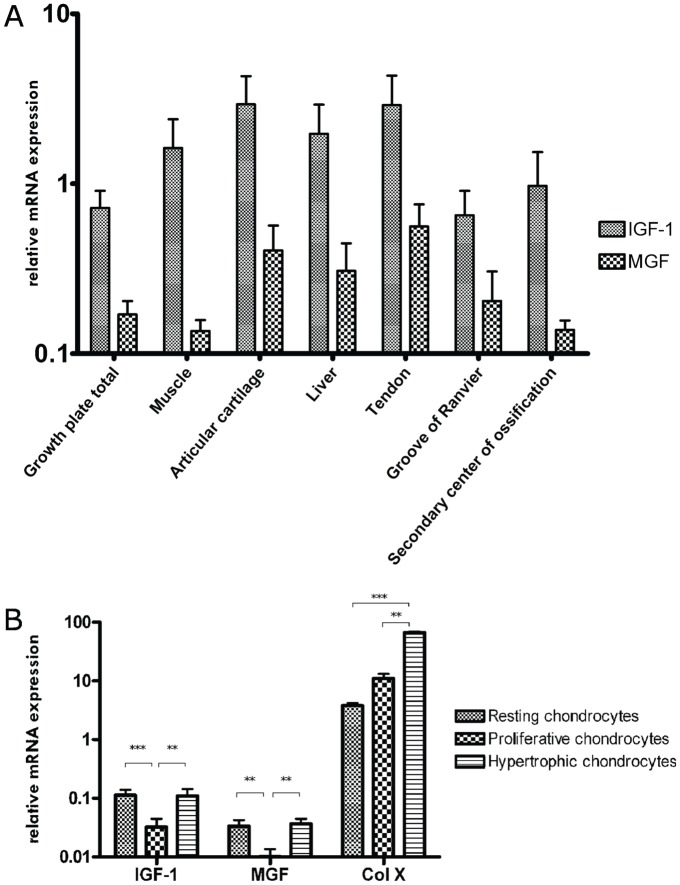
*Mgf* and *Igf1* mRNA expression profile (normalized to 18S rRNA) of various porcine tissue samples (A) and within different zones of the growth plate separated by laser capture microdissection (B) as quantified by real-time PCR. (n≥3 per tissue, One-way ANOVA of log-transformed data (a) or Kruskal-Wallis with Bonferroni-adjusted post-hoc tests (b) were used to detect differences between groups: ** p<0.01; ***p<0.001 vs proliferative chondrocytes; ^###^p<0.001 vs hypertrophic chondrocytes. Data are presented as mean + SEM).

Real-time PCR was applied to compare *Mgf* and *Igf1* expression in different porcine tissues. Neither *Mgf* (F_6,25_ = 1.11, p = 0.385, η^2^ = 0.210) nor *Igf1* (F_6,24_ = 1.24, p = 0.322, η^2^ = 0.237) expression differed between the growth plate, skeletal muscle, liver, articular cartilage, tendon, groove of Ranvier and secondary center of ossification (Fig. 3A). Comparison of mRNA expression revealed that the *Mgf* fraction of total *Igf1* ratios varied between the tissues examined ranging from 8% in muscle to 31% in Ranvier's groove (variance not statistically significant). No expression of *Mgf* could be found in brain tissue (hippocampal regions, data not shown).

In addition to *Mgf* and *Igf1*, the expression of *collagen-10a1* was determined to control the separation of the different zones in laser microdissected samples. The Kruskal-Wallis test revealed statistically significant differences between different zones of the growth plate for *Igf1* (H_2_ = 12.3, p = 0.002) and *Mgf* (H_2_ = 10.3, p = 0.006), and Bonferroni-adjusted, post-hoc tests showed that *Igf1* and *Mgf* expressions were lower in the proliferative zone than in the resting (p<0.01) and hypertrophic zones (p<0.01, Fig. 3B). As in published data, *collagen-a10a1* expression could be detected in all three zones, although to different extents (H_2_ = 30.8, p = 0.008) [42]. The expression was higher in the hypertrophic zone than in the resting (p<0.001) and proliferative zones (p<0.001). Comparing mRNA expression, *Mgf* was found to represent approximately one third of the overall *Igf1* expression in all three zones of the growth plate. Primer efficiency was nearly similar for *Mgf* and *Igf1* primers (data not shown) [46].

### Immunohistochemistry of MGF

**Figure 4:**
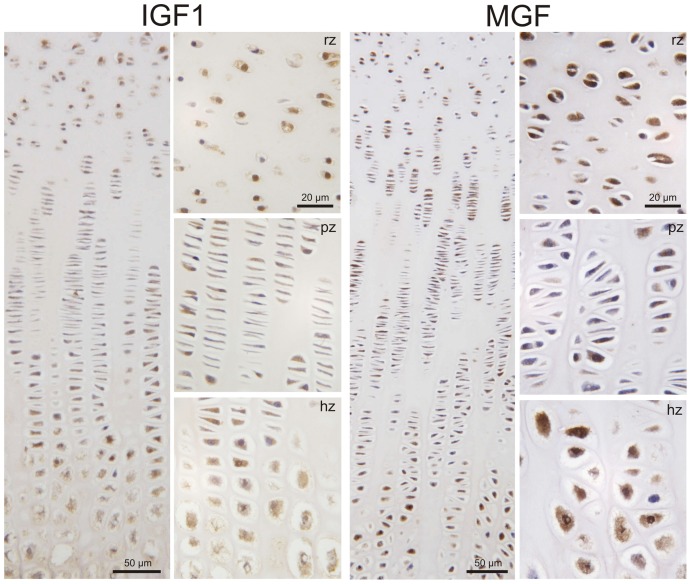
Immunohistochemical detection of MGF and IGF-1 in the porcine growth plate (overview, magnification 10x). MGF and IGF-1 was detected in chondrocytes of the resting (rz), proliferating (pz) and hypertrophic (hz) zone of porcine growth plate. We found the MGF peptide in the cytoplasm and in some chondrocytes also in the nucleus of resting and hypertrophic chondrocytes, although some cells were completely negative. More negative cells were detected in the proliferating zone (magnification 50×).

**Figure 5:**
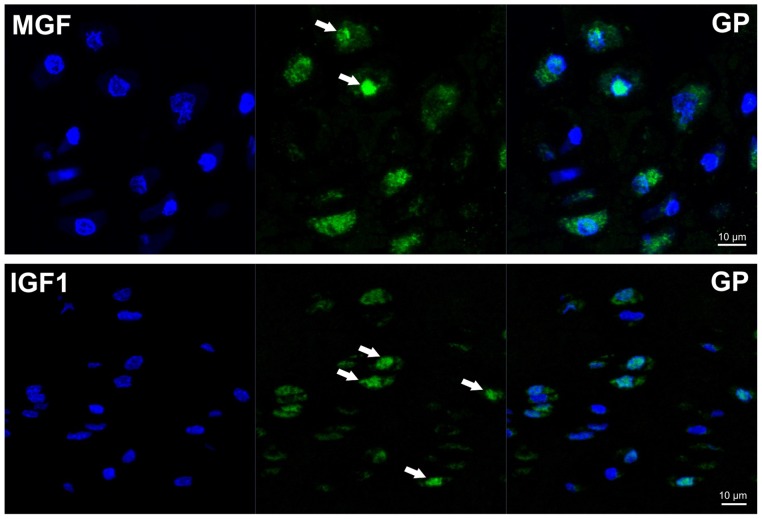
Immunofluorescent staining of GP cartilage (rz) for MGF and IGF1 shows co-localisation of green signal (white arrows) with DAPI stained nuclei (blue) in few cells. Some cells are negative for MGF as well as IGF1.

We detected MGF protein in the growth plate in a distinct distribution. Most of the chondrocytes were strongly stained in the resting zone of 3 to 6-week-old piglets, whereas the flat cells in the proliferating zone only rarely expressed MGF. Expression of MGF was frequently found in the chondrocytes of the prehypertrophic and hypertrophic zones, similar to the IGF-1 staining in the growth plate (Fig. 4). In the MGF positive cells, staining was found in the cytoplasm and in some chondrocytes also in the nucleus (Fig. 5).

**Figure 6:**
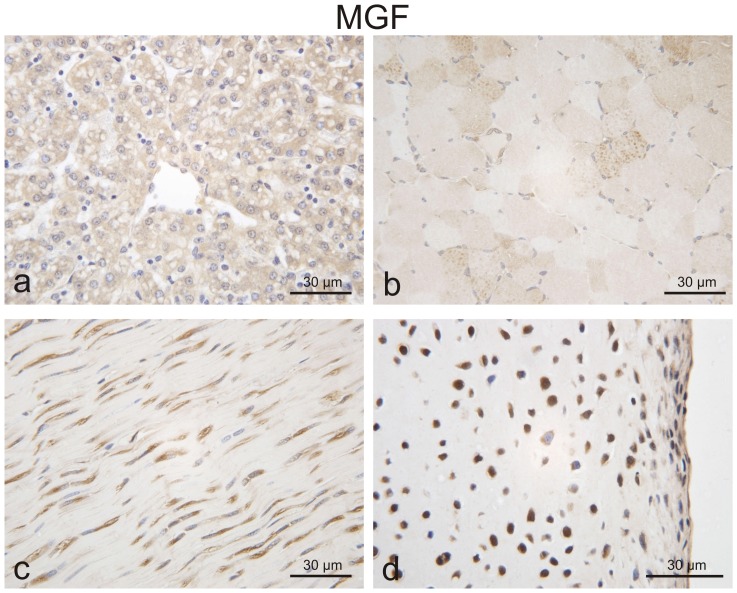
Immunohistochemical detection of MGF in porcine liver (a) tendon (c) and articular cartilage (d). Only weak staining was seen in porcine skeletal muscle (b), (magnification 40×).

MGF was not only expressed in growth plates, as we could also detect the peptide in articular cartilage and in non-chondrogenous tissues, like liver and tendons. Interestingly, we could detect weak MGF staining in unstimulated muscle (Fig. 6).

### Effect of MGF and IGF-1 on growth plate chondrocytes

**Figure 7:**
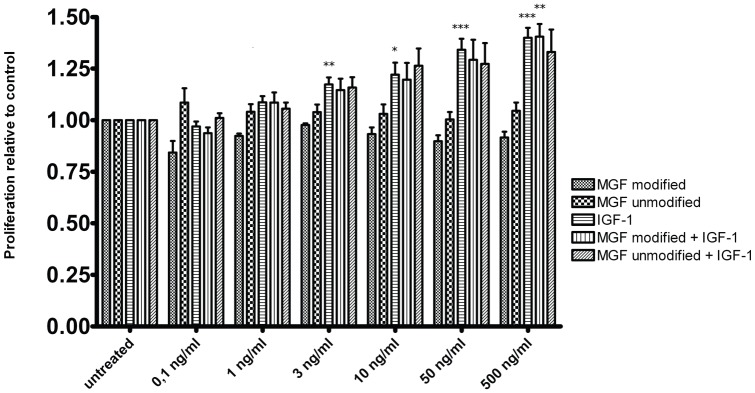
Proliferation effects of MGF/IGF-1. Effects of IGF-1, different MGF peptides and their combination on proliferation of porcine growth plate chondrocytes as measured by BrdU staining. (n = 5–11 per group; One-way ANOVA of log-transformed data, Games-Howell post-hoc analyses: * p<0.05; ** p<0.01; *** p<0.001 vs respective untreated control; Data are shown as mean + SEM).

We did not observe any statistically significant effects of the MGF peptides on the proliferation of monolayer growth plate chondrocytes in concentrations ranging from 0.1 to 500 ng/ml. However, differences between concentrations could be detected when growth plate explants were treated with IGF-1 alone (F_6,70_ = 18.1, p = 0.000, η^2^ = 0.608) (Fig. 7). Games-Howell post-hoc analyses revealed a dose-dependent increase of proliferation in explants treated with 3 (p = 0.004), 10 (p = 0.033), 50 (p = 0.000), and 500 ng/ml IGF-1 (p = 0.000) compared with untreated control. This trend towards a proliferative effect was also observed when MGF peptides were added in combination with IGF-1 (modified MGF + IGF-1: F_6,28_ = 7.8, p = 0.000, η^2^ = 0.626; unmodified MGF + IGF-1: F_6,35_ = 3.8, p = 0.005, η^2^ = 0.395), but post-hoc analyses revealed that differences were only statistically significant between the highest concentrations of modified MGF with IGF-1 (500 ng/ml) and the untreated control (p = 0.010). Combined MGF and IGF-1 treatments showed no statistical difference in comparison to IGF-1 treatment alone.

## Discussion

### Expression pattern of MGF

Normal longitudinal growth in the fetus as well as in postnatal life is achieved by the coordinated recruitment, proliferation, hypertrophy and apoptosis of chondrocytes within the epiphyseal growth plate of long bones and the spine. This extremely complex process is orchestrated by endocrine, paracrine, autocrine and even intracrine regulation, and involves complex local signaling loops and systemic metabolic, nutritional and classical endocrine pathways [47], [48], [49]. All these factors influence gene expression and power output by hypertrophy and augmentation of tissue mass. MGF, a splice variant of IGF-1, is one autocrine factor that has been found to play an important role in tissue, namely in muscle, hypertrophy. In muscle, mechanical exercise or damage leads to the synthesis of *Igf1* mRNA, which is first spliced towards *Mgf* [23], [50]. Other *Igf1* isoforms usually appear some days later at the expense of the *Mgf* isoform. *Mgf* has been described as a factor which “kick starts” the proliferation process [51].

To our knowledge, up until now MGF has not been reported in the porcine genome. We screened porcine EST-data bases, compared the sequences with the human *Mgf* sequence and discovered that the sequence had already been analyzed but not recognized as *Mgf*.

We designed specific primers, which helped us to perform quantitative analyses of the *Mgf* mRNA expression level as well as the total expression level of *Igf1* mRNA in porcine growth plates.

Our data show that *Mgf* and *Igf1* mRNA can be differentially detected in the porcine growth plate and the *Mgf* splice variant distinguished from the systemic *Igf1-Ea* variant. When we looked at the distribution of *Mgf* mRNA within the different zones of the growth plate, we found the highest expression levels in the resting and hypertrophic zones. Immunohistochemistry, although a mainly qualitative method, showed similar results. *Igf1* and *Mgf* were also expressed in notable amounts in the groove of Ranvier and the secondary center of ossification, which emphasizes these chrondo-osseous tissues' contribution to endochondral bone development. Only low expression of *Mgf* mRNA was detected in unstimulated muscle and none in brain tissues (hippocampal regions, data not shown), where *Mgf* has been found after ischemic periods within these tissues [20].

### Influence of MGF on proliferation

Proliferation may be achieved by the C-terminus itself, as the carboxy-terminus has been suggested to function as an independent biologically active peptide [52]. Myoblasts have been found to increase in number but remain mononucleated cells when muscle stem cells are transfected with *Mgf* cDNA or treated with its carboxy-terminal peptide [23]. Blocking the IGF-1 receptor (IGF-1R) did not influence the outcome of the experiment. More recent studies in several other cell types have consistently confirmed that MGF-mediated effects are not dependent on the IGF-1R [20], [53], [54].

The IGF-1R mediates two signaling pathways, namely the Raf-MEK-ERK pathway, inducing proliferation and the PI3K-Akt pathway, supporting differentiation [55], [56]. Hypertrophy in muscle cells has been described to be mediated by IGF-1R-dependent activation of the Akt pathway [57]. In contrast to mature IGF-1, MGF has been reported to activate ERK1/2 without influencing Akt phosphorylation [56]. However, which extracellular receptor mediates the effect of MGF remains unclear.

When we performed proliferation experiments with cells from the growth plate, we were able to stimulate proliferation of cells from the resting and proliferation zones with IGF-1 at concentrations of 50 ng/ml and 500 ng/ml.

This raised the question of whether proliferation of growth plate chondrocytes could also be achieved by MGF, as has been described for other cells. Our stimulation experiments showed that in contrast to IGF-1, both an unmodified and a modified MGF peptide with increased stability failed to stimulate proliferation in growth plate cells. When primary chondrocytes were co-stimulated with IGF-1 and MGF, the proliferation rates did not differ statistically significantly from those achieved by stimulation with IGF-1 alone (Fig. 7). Our experiments suggest that MGF added exogenously does not influence the proliferation behaviour of growth plate chondrocytes. This is at variance with proliferation results from groups investigating other tissues but it is plausible that MGF has different modes of action in different tissues [20], [23], [53].

Early work on MGF in muscle tissue described this form of the *Igf1* gene as being unglycosylated and probably having a shorter half-life time. Therefore, MGF was assumed to be designated for an autocrine rather than a systemic mode of action [19]. Furthermore, MGF has been shown not to stimulate proliferation in other tissues, like lens epithelial cells [58]. Based on these data, proliferation by exogenous MGF stimulation, as has been described in the other studies mentioned above, is rather unexpected.

### Localization of the MGF peptide within the cell

We found MGF to be mainly localized around and also within the nucleus of cells. Therefore, we speculate that the MGF peptide is directly involved in transcriptional processes. Similar localizations of MGF have been described in experiments in HeLa cells using IGF exons tagged with green fluorescent protein [59]. Furthermore, a clear nuclear localization signal “RRRK” within the Ec peptide has been observed when using the PROST II program for sequence comparison [60].

MGF does not bind to IGF-1 binding proteins, which are responsible for stabilization of IGF-1 in blood and in muscle, at least not to any that are known. Functional epitope mapping has shown that IGF binding proteins mainly interact with the residues 1–3 and 49–51 and the IGF-1 receptor interacts with the residues 21, 23, 24, 44 as well as the tyrosines 31 and 60, which are located in the protein domains C and A [61]. All these epitopes are encoded by exons 3 and 4 of the *Igf* gene. When postulating that the Ec region acts independently of the rest of the protein and is functional on its own, the fact that MGF has no binding site for any known IGF binding protein has to be taken into account. Additionally, the peptide is reported to be unglycosylated. Therefore, it is unlikely that MGF can act in a systemic mode of action. Furthermore, the MGF molecule features no epitope for interaction with the IGF-1 receptor, which is also encoded by exons 3 and 4. Neutralizing antibodies against the IGF receptor have been shown to block the effect of IGF-1, but not that of MGF [20], [53], [54], [56].

MGF can be regarded as an autocrine factor with an extremely short half-life outside the nuclear environment [19], [27], [59]. We favour the idea that MGF is an intracellular peptide with a clear nuclear localization signal, and probably features a function in the transcriptional process. This would explain why exogenously added MGF showed no effect on proliferation.

The insulin-like growth factor system is a phylogenetically ancient family of peptides involved in a variety of actions like growth, differentiation, cell migration, survival, senescence or cell motility, carcinogenesis and adhesion. Many of these effects have been attributed to IGF-1 or its receptors [18], [62], [63], [64], [65], [66], [67]. We show that MGF represents approximately one third of all IGF-1 isoforms expressed within the growth plate. However, at least in our hands, MGF does not contribute to proliferative effects on porcine chondrocytes when added exogenously.

There seem to be two hypotheses of the way in which MGF acts. One theory is that MGF acts via the ERK pathway. This hypothesis is supported by the successful cell proliferation and phosphorylation of pERK1/2 with exogenously added MGF [56]. The other theory is that MGF is a peptide that acts intracellular, probably within the nucleus. Histological findings, IGF receptor independent effects, lack of protein stabilizing structures and a nuclear localization signal as well as our results favor this theory. On the protein level, we found MGF in several tissues within the nucleus. Interestingly, the smallest amount of MGF within the growth plate was detected in proliferative chondrocytes, which have been shown to be responsible for approximately 10% of total linear growth [68]. In comparison to previous studies in other tissues, we did not achieve any proliferative effects of growth plate chondrocytes when we added MGF exogenously. In our opinion, this is not so surprising but rather likely, considering MGF has no sites for IGF binding proteins or the IGF receptor. Based on our results, we conclude that this family member is located in the cytoplasm and around the nucleus. Whatever function this peptide has to fulfil within the nucleus remains to be determined.

## Supporting Information

Figure S1 **Negative controls were prepared by omission of the primary antibody followed by the secondary system and in addition by pre-incubation of the IGF1 and MGF antibodies with the respective blocking protein.**
(TIFF)
