# Supplementary figures and images for: Insulin-Like Growth Factor I (IGF-1) Ec/Mechano Growth Factor – A Splice Variant of IGF-1 within the Growth Plate
Source: PLoS One. 2013 Oct 11;8(10):e76133. doi: 10.1371/journal.pone.0076133 (PMC3795771; doi:10.1371/journal.pone.0076133)

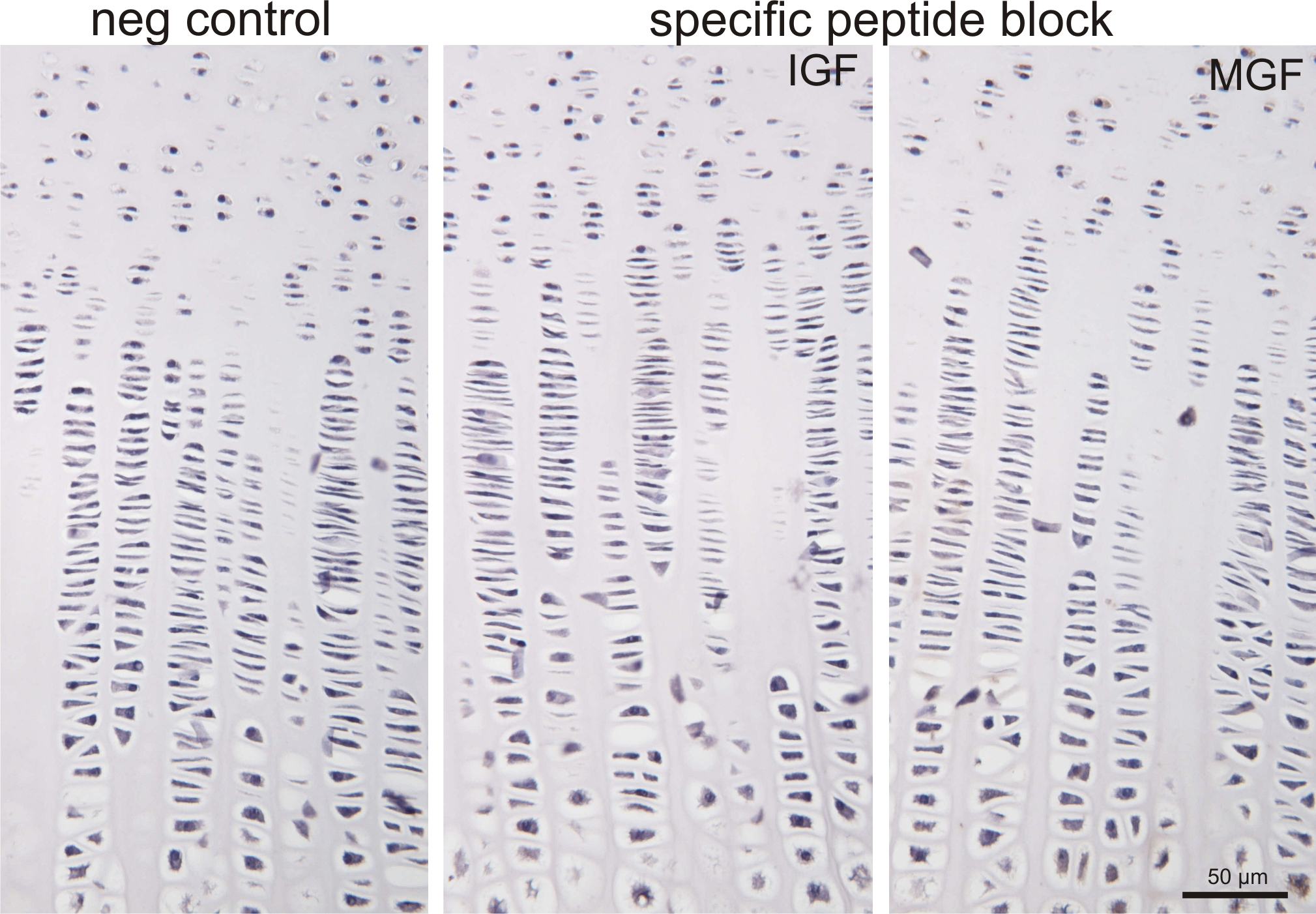

Supplement: Figure S1 — Negative controls were prepared by omission of the primary antibody followed by the secondary system and in addition by pre-incubation of the IGF1 and MGF antibodies with the respective blocking protein. (TIFF) [file pone.0076133.s001.tiff]
